# Burden of childhood diarrhea and cholera outbreaks in the Lake Tana Basin (Ethiopia): Review

**DOI:** 10.4314/ahs.v24i3.34

**Published:** 2024-09

**Authors:** Bayeh Abera

**Affiliations:** Medical Microbiology, Department of Medical Microbiology, College of Medicine and Health Sciences, Bahir Dar University, Ethiopia

**Keywords:** Cholera, diarrhoea, climate change, Lake Tana Basin, Ethiopia

## Abstract

**Objectives:**

Diarrhoea is one of the leading causes of childhood morbidity and mortality, while cholera outbreaks are a major public health emergency insub-Saharan African countries. This study was aimed at investigating the link between climate changes and cholera outbreaks, and the burden of childhood diarrhoea in the Lake Tana Basin (Ethiopia).

**Methods:**

Research articles published in English were searched from Google scholar, PubMed, and Web of science and these were supplemented by a four-year secondary data of childhood diarrhoea and cholera outbreaks extracted from health management information system (HMIS) in 61 districts.

**Results:**

The mean prevalence of diarrhoea per 1000 children was 420 (95% CI:311.7; 528.6). The prevalence of childhood diarrhoea showed spatial and temporal variation hereby 16.4% (10/61) districts exhibited high prevalence (>201/1000 children). The prevalence of diarrhoea was significantly higher in boys compared to girls (p = 0.001), while the incidence of cholera was significantly higher in females compared to males (p < 0.001). Heavy precipitation and El-Niño events were linked to the episodes of cholera outbreaks.

**Conclusions:**

Health interventions should consider spatial and temporal variations of diarrhoea. The cholera outbreak preparedness needs to be aware during the events of climate changes in the hotspot areas.

## Introduction

Diarrhoea is the major public health problem worldwide particularly in sub-Saharan African countries related to poor sanitation and lack of safe water supplies. Diarrhoea and cholera are caused by pathogenic microorganisms by the ingestion of faecally contaminated drinking water and food. Cholera is a bacterial disease caused by Vibrio cholerae and it causes severe acute rice water diarrhoea and dehydration, while diarrhoea is a symptom of intestinal infection caused by different types of pathogens of bacteria, viruses, and protozoa.^1^-^3^ Childhood diarrhoea is the leading cause of death in children under five years old and accountable for the mortality of 370,000 children in 2019. 4 In sub-Saharan African countries, mortality caused by diarrhoea reached 37% of all deaths during the first year of life. 5 Likewise, cholera was responsible for 2.9 million cases and over 95,000 deaths in endemic countries.^6^ Ethiopia has high childhood morbidity and mortality linked to childhood diarrhoea. Studies conducted at community levels in the Lake Tana Basin reported that a two-week prevalence of childhood diarrhoea was 28.8 - 36.1%. 7, 8 Multiple risk factors had been associated with the prevalence of diarrhoea. Poor sanitation, unsafe water supplies, poor handwashing habits. 7, 8 and climate conditions (eg., temperature and rainfall) are the major risk factors contributing to the spread of diarrhoea and cholera outbreaks.^9^-^11^ Studies showed that climate changes such as heavy rainfall, floods, elevated temperature can influence the microbial quality of drinking water and enhance the risk of exposure to diarrhoea and cholera.^12^, ^13^

The epidemiology of diarrhoea and cholera epidemics would be changed owing to overcrowding human population, poverty, urban chaos, and climate changes. Moreover, Ethiopia, the actual burden of cholera is not known because of several factors.^14^ The prevailing data on the impacts of climate conditions on cholera outbreaks at regional and local levels are essential to evaluate and plan interventions measures. In addition, Lake Tana Basin is the largest freshwater body in the country, which have favorable condition for transmission of diarrhoea as annual flooding occurs in the districts near the northeast lakeshore, which can discharge faecal contaminates into waterbodies. Lake Tana and its tributary rivers are used as a direct and indirect sources of water supply for drinking water for the people living near the lake shores. 15 Thus, the objectives of this systematic review were: i) to summarize the burden of childhood diarrhoea and identify the hotspot districts for childhood diarrhoea prevalence in the Lake Tana Basin using literature review and a four-year secondary data; ii) to explain why cholera outbreak has been a recent phenomenon since 2006 in the catchments of Lake Tana Basin; iii) to explore risk factors contributing to diarrhoea and chorea outbreaks.

## Methods

### Study area

**Figure 1:**
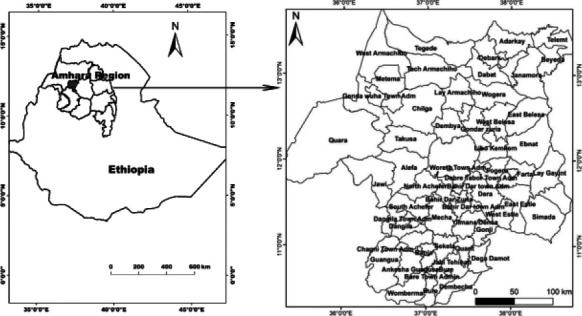
Map of the study area (Lake Tana Basin); **Left**: map of the Ethiopia showing the Lake Tana Basin. **Right**: districts of the Lake Tana Basin where secondary data analysis and literature review were conducted

The Lake Tana Basin (LTB) is located in northwestern Ethiopia, and it has an area of 15,000 km^2^ and 20% of this area is covered by the Lake (Figure 1). A total of 61 districts (Woredas) were included from the administrative zones of Bahir Dar Zuria, Awi zone, North, Central, West and South Gondar zones, and West Gojjam zone. In the LTB, more than three million people live, of which 85% are farmers. The climate of the LTB is dominated by tropical highland monsoon with most of its rainfall (70-90% of total rainfall) occurring between June and September.^16^

### Data sources and synthesis

This study used two strategies for the acquisition of data on diarrhoea and cholera cases. First, a literature review was conducted on studies conducted on childhood diarrhea and cholera in the Lake Tana Basin. Second, four-year childhood diarrhoea and cholera data were obtained from Amhara Regional National State Health Bureau.

### Inclusion and exclusion criteria

**Figure 2:**
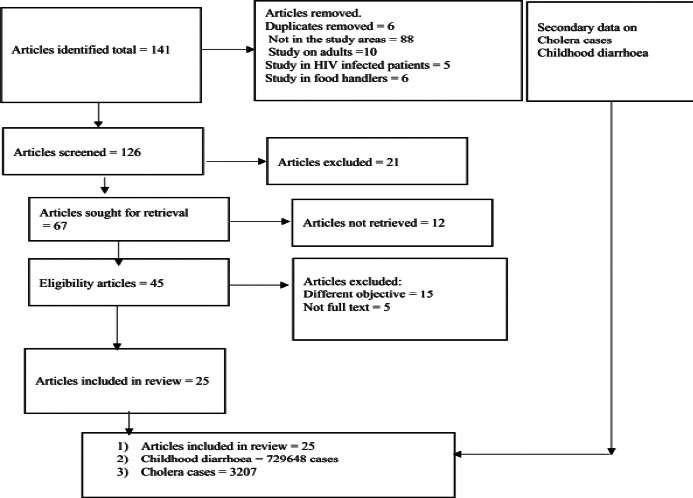
The PRISAM flow diagram for articles included, exclude and a four-year retrospective childhood diarrhoea and cholera data from health facilities in the catchments of the Lake Tana Basin

In this review the inclusion criteria were research articles and gray literature published on diarrhoea among children under five years of age, cholera cases in all age groups, any years of publication, articles published in English, study area (Lake Tana Basin), while studies that did include the study area and studies included adult diarrhoea were excluded based adopting the Preferred Reporting Items for Systematic Reviews and Meta-Analysis (PRISMA) flow char (Figure 2).^17^

### Literature review and data items

The review was conducted based on the PRISMA guideline. A literature search was conducted using PubMed, Google Scholar and Web of science data bases using the key words based on Boolean operator: diarrhoea, cholera, acute watery diarrhoea, *Entamoeba histolytica, Giardia lamblia, Cryptosporidium parvum*, and *Isospora belli, Shigella spp., Escherichia coli* O157, and *Campylobacter jejuni, rotavirus, adenovirus*, and *norovirus*. From included articles, data such as etiologies of diarrhoea, year of publication, sample size, prevalence, risk factors and laboratory methods were extracted. In addition, cholera outbreak reports were searched from weekly bulletins of the World Health Organization (WHO) and Amhara Public Health Institute (APHI).

### Secondary data

It was anticipated that studies were not inclusive for all districts of the study areas hence tit was supplemented by a four-year retrospective data on childhood diarrhoea and cholera cases from health care services. A four-year secondary data on childhood diarrhoea and cholera cases (July 01, 2014, to June 30, 2017) was obtained from Amhara Regional Health Bureau through the Health Management Information System (HMIS). The relevant data such as age, sex, quarters, and years of report and districts were extracted. Simultaneously, the projected populations of districts were obtained from the Amhara Regional State Bureau of Finance and Economic Development. The health care facilities recorded childhood diarrhoea cases per the World Health Organization guidelines. Therefore, childhood diarrhoea is defined as the passage of three or more loose or liquid stools per day in children under five years of age.^4^

### Statistical analyses

The annual childhood diarrhoea prevalence per 1000 people at risk was calculated using the projected annual population for under five children for each district, while cholera incidence was calculated per 100000 people at risk. A chi-square test was done to determine the association between gender and cholera cases. P-value < 0.05 with 95% confidence interval (CI) was considered as statistically significant.

### Geospatial mapping of childhood diarrhoea

The retrospective childhood diarrhoea data from 61 districts were clustered as prevalence per 1000 children as follows: prevalence of diarrhoea <100/1000 children was clustered as low prevalence; Prevalence of diarrhoea 101-200/1000 as moderate prevalence and ≥ 201/1000 as high prevalence. Then, the values were geo-coded using Arc Map 10.1 raster classification tools. Similarly, altitude geo-spatially referenced maps were developed to link the relationships between clusters of diarrhoea with altitude.

## Results

### Articles published on diarrhoea and cholera

A total of 141 research articles were identified. Of these, 25 articles satisfied the inclusion criteria for synthesis (Figure 2). All research articles included in this review were published over a period of 21 years (2001 to 2022). Among 25 studies, 13 studies identified the causative microorganism of diarrhoea, while 12 articles studied the prevalence of childhood diarrhoea based on interview using questionnaires (Table 1). In this review, two studies used a case control design to identify attributable risk factors for childhood diarrhoea.

**Table 1:** Articles included for review of childhood dirrhoea and cholera outbreaks in the Lake Tana Basin

References	Year	Study areas	Data collection tool	Sample size	Prevalence in %
15	2001	North Gondar	Questionnaire	1101	18
16	2010	North Gondar	Questionnaire	440	Case control
18	2010	West Gondar	Laboratory	81	Outbreak
19	2011	West Gojjam	Questionnaire	768	18
20	2011	Bahir Dar	Laboratory	215	14.9
21	2013	North Gondar	Laboratory	410	62.2
22	2013	North Gondar	Laboratory	304	34.2
23	2013	South Gondar	Laboratory	2338	11 to 23
24	2013	North Gondar	Laboratory	285	15.4
25	2015	Bahir Dar	Questionnaire	667	21.6
26	2015	Bahir Dar	Laboratory	422	7.9 to 9.5
27	2015	Bahir Dar	Laboratory	422	48.3
28	2016	Bahir Dar	Laboratory	394	55
29	2017	West Gojjam	Questionnaire	775	21.5
30	2017	Bahir Dar	Questionnaire	715	20
31	1018	North Gondar	Questionnaire	736	22.1
32	2018	Bahir Dar	Questionnaire	553	9.4
8	2018	Awi	Questionnaire	525	9.9 – 36.1
33	2018	West Gojjam	Questionnaire	351	Case control
34	2019	Bahir Dar	Questionnaire	498	14.8
1	2020	Bahir Dar	Laboratory	344	47.1
7	2021	Awi	Questionnaire	419	25.3
35	2022	Bahir Dar	Laboratory	144	Na
36	2019	Bahir Dar	Laboratory	450	32
37	2022	South Gondar	Laboratory	38	52.6

### Aetiology of diarrhoea

Studies reported that among protozoan parasites, *Giardia lamblia* and *Cryptosporidium parvum* were the most reported aetiologies of diarrhoea. For bacterial diarrhoea, *Shigella spp., Escherichia coli* O157, and *Campylobacter jejuni*, were commonly reported. For enteric viral agents of diarrhoea, Adenovirus, Norovirus and Rotavirus were documented (Table 2).

**Table 2:** The identified aetiology of diarrhea with prevalence% in the Lake Tana Basin

Aetiology of diarrhea	Prevalence %	References
**Protozoa**		
*Entamoeba histolytica*	14.1 to 15.2	38, 39
*Giardia lamblia*	23.4 to 55.0	23, 28
*Cryptosporidium parvum*	12.8 to 43.6	1, 28

**Bacteria**		
*Shigella* species	14.9 to 15.6	20
*Escherichia coli* O157	10.2 to 28.9	1
*Campylobacter jejuni*	15.4	24
*Salmonella spp*.	1.7	1

**Virus**		
Adenovirus	18.4 to 32	36,37
Astrovirus	3.6 to 5.3	36,37
Noroviruses	13.2	37
Rotavirus	10.5	37

### Burden of childhood diarrhoea

**Figure 3:**
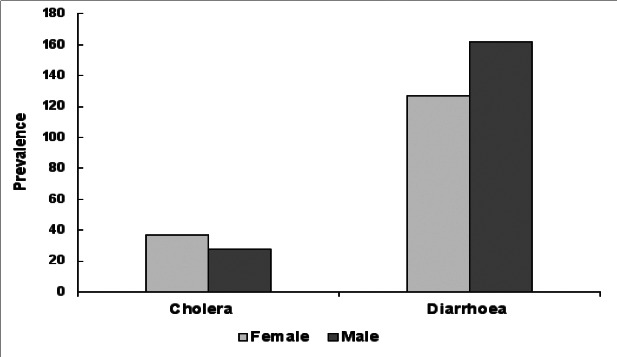
The incidence of cholera outbreaks and prevalence of childhood diarrhoea in relation to gender. Childhood diarrhoea prevalence was significantly higher in male children compared to females [(COR= 1.3 (1.32-1.4); p < 0.001] and cholera outbreaks was significantly higher in females compared to males [(COR=0.76 (7.1 to 0.81; p < 0.001)

**Figure 4:**
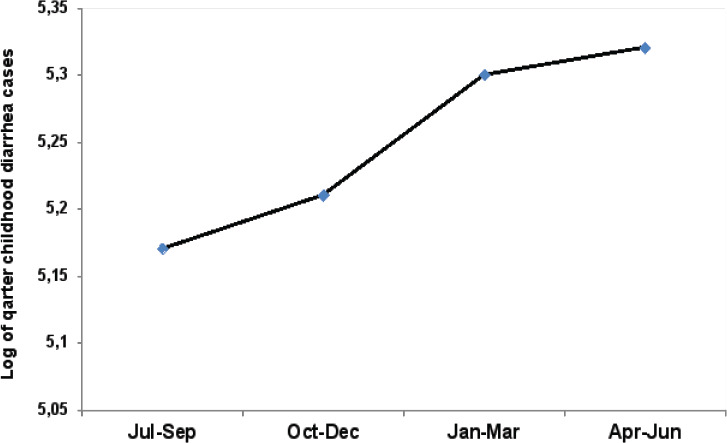
The overall prevalence of childhood diarrhea based on the quarter reports

**Figure 5:**
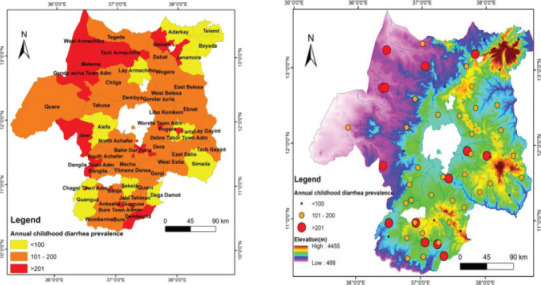
Geospatial map of districts with prevalence of childhood diarrhea in Lake Tana Basin. **Left**: name of districts with prevalence of childhood diarrhea; **Right**: prevalence of childhood diarrhea with altitudes of districts. Prevalence of diarrhea <100 = low prevalence; Prevalence of diarrhea 101-200 = moderate prevalence and ≥ 201 = high prevalence

The overall annual mean prevalence of childhood diarrhoea per 1000 children was 420 (95%CI: 311.7, 528.1). The prevalence of diarrhoea was higher in males (161.8/1000 children) compared to female (126.9/1000 children) (p < 0.001) (Figure 3). The assessment of seasonal trends showed that in each year, childhood diarrhea increased at the beginning of rainy months and in dry months (Figure 4). Furthermore, the prevalence of diarrhoea demonstrated geospatial variation. Among 61 districts, 16.4% (10/61) exhibited a high prevalence of childhood diarrhea (≥ 201/1000) (Figure 5). The associated risk factors contributing to diarrhoea were unsafe drinking water supplies (AOR = 2.59) and the lack of access to latrine (AOR = 3.00). The detailed prevalence of childhood diarrhoea from 61 districts are displayed (supplementary Table 1).

**Table S1:** prevalence of childhood diarrhea per 1000 population at district level in the Lake Tana Basin from 2014-2017

Name of districts	Zones	2014	2015	2016	2017	Average
Dangila Zuria	Awi	77.7	87.0	117.9	153.9	109.1
Guagusa Shikudad	Awi	75.4	188.7	170.7	166.9	150.4
Fagita Lekoma	Awi	91.4	112.7	144.7	169.8	129.7
Zigem	Awi	116.5	177.4	170.4	241.7	176.5
Injebara	Awi	194.7	185.4	246.4	282.6	227.3
Ankesha Guagusa	Awi	139.0	90.3	121.1	94.6	111.3
Jawi	Awi	175.1	185.1	269.6	293.6	230.9
Chagni	Awi	669.7	400.3	411.7	449.4	482.8
Guangua	Awi	77.6	79.9	87.3	110.6	88.8
Banja	Awi	95.7	108.7	149.1	194.0	136.9
Dangilla Town	Awi	116.8	161.5	191.4	234.9	176.1
Degadamot	West Gojjam	73.8	82.7	113.1	113.1	95.7
Dembecha	West Gojjam	119.0	220.4	279.4	334.0	238.2
Quarit	West Gojjam	62.3	111.1	109.6	121.6	101.1
F.Selam Town	West Gojjam	169.9	151.7	354.7	414.7	272.8
Jabitehnan	West Gojjam	118.7	133.6	156.0	166.5	143.7
Wonberma	West Gojjam	95.6	136.1	180.6	236.0	162.1
Burie Town	West Gojjam	85.8	253.3	265.9	345.0	237.5
Burie Zuria	West Gojjam	70.8	125.9	174.0	189.9	140.1
Sekela	West Gojjam	64.3	67.8	94.3	142.9	92.3
Semen Achefer	West Gojjam	68.5	89.7	108.2	138.0	101.1
Debub Achefer	West Gojjam	159.5	189.8	260.0	174.0	195.8
Mecha	West Gojjam	116.6	158.0	202.3	196.3	168.3
Yilimana Densa	West Gojjam	61.4	123.3	142.1	152.5	119.8
Gonji Kolela	West Gojjam	75.6	133.9	164.5	280.0	163.5
Bahirdar Zuria	Bahir dar zone	97.8	123.1	208.6	241.2	167.6
Bahir Dar Town	Bahir dar zone	502.3	749.9	558.3	516.1	581.6
Adi Arkay	North Gondar	57.0	75.1	115.1	138.9	96.5
Alefa	North Gondar	42.2	98.1	106.0	110.9	89.3
Beyeda	North Gondar	46.6	66.9	89.1	98.9	75.4
Chilliga	North Gondar	140.4	128.1	126.8	156.4	137.9
Dabat	North Gondar	82.8	154.8	101.5	139.3	119.6
Debark Zuria	North Gondar	88.5	101.9	745.4	95.3	257.8
Dembia	North Gondar	66.0	87.7	128.0	128.3	102.5
Gendewuha	North Gondar	289.4	367.5	631.5	632.0	480.1
Janamora	North Gondar	33.4	38.2	68.1	72.4	53.0
Gondar Zuria	North Gondar	109.5	106.8	122.5	174.1	128.2
Lay Armachiho	North Gondar	56.9	40.7	63.4	89.2	62.5
Metema	North Gondar	182.6	259.2	274.6	205.9	230.6
Quara	North Gondar	122.3	224.2	183.5	244.5	193.6
Tach Armachiho	North Gondar	114.3	186.8	271.3	273.1	211.4
Takusa	North Gondar	86.0	128.7	103.5	106.2	106.1
Tegedie	North Gondar	83.7	267.8	101.5	145.9	149.7
Telemet	North Gondar	45.7	65.0	141.9	131.3	96.0
Mirab Armachiho	North Gondar	284.3	562.6	399.3	414.9	415.3
Wogera	North Gondar	64.5	92.6	140.6	106.7	101.1
Misrak Belesa	North Gondar	79.2	85.8	148.8	180.0	123.4
Debark	North Gondar	90.8	84.2	26.0	182.4	95.9
Mirab Belessa	North Gondar	152.9	168.1	179.2	208.0	177.0
Fogera	South Gondar	143.8	159.9	184.9	281.6	192.5
Mirabe Estie	South Gondar	87.0	92.8	140.0	107.0	106.9
Misrak Estie	South Gondar	81.4	113.0	192.3	165.3	138.0
Woreta	South Gondar	619.0	526.1	475.4	561.8	545.6
Dera	South Gondar	87.5	147.4	137.3	171.7	136.0
Libokemkem	South Gondar	118.4	151.6	171.7	227.0	167.2
Lay Gayint	South Gondar	72.2	113.8	155.2	138.0	119.8
Farta	South Gondar	68.6	107.0	121.3	148.3	111.3
Tach Gayint	South Gondar	59.6	117.2	124.0	155.6	114.1
Ebenat	South Gondar	101.9	97.8	120.4	130.6	112.7
Simada	South Gondar	64.7	75.6	107.3	115.0	90.6
Debre tabor Town	South Gondar	295.3	265.9	251.9	284.4	274.4

### Cholera outbreaks

Cholera has been considered as a recent phenomenon in the catchments of Lake Tana Basin. Records showed that a cholera outbreak occurred for the first time in August 2006 in West Gondar administrative zone in Metema district. The Vibrio cholerae 01 sero type Inaba was the aetiology of this outbreak.^18^ The second cholera epidemic occurred in 2014 from June to September. A four-year data analysis showed that 3207 cholera cases were recorded in 2014, 2016 and 2017. The overall incidence of cholera per 100000 people at risk was higher in females compared to males (Figure 2) (36.7 verses and 27.9) with (crude odds ratio = 0.76, p <. During the first cholera outbreak in 2006, heavy rainfall (100-150% of the normal precipitation) was recorded in the Lake Tana Basin. Likewise, the second cholera outbreak started in June 2016 following the incidence of El-Niño (2015 to 2016) in Ethiopia. Table 3 shows the detailed cholera cases linked to climatic conditions.

**Table 3:** The association of cholera outbreaks with climate conditions in the Lake Tana Basin

Years	Cases	Climate data	Reference
**Aug-Sep 2006**	13386, CFR = 1.6	150% increase in rainfall of the normal	40, 41
**June-Sep 2016**	3565; CFR = 0.8	Following El-Niño 2015	42, 43
**Jun-Aug 2017**	3207	Following El-Niño 2015-2016	42

Key: CFR: case fatality rate

## Discussion

This systematic review showed that data on prevalence of pathogen specific diarrhoea are limited due to lack of laboratory settings. However, a few studies identified the common causative agents of the enteric protozoa,^23^, ^28^, ^38^, ^39^; pathogenic bacteria 27, and viruses.^36^, ^37^. A four-year data analysis showed that childhood diarrhoea prevalence was consistent for three years. However, the prevalence declined sharply in 2017. The reason might be related to interventions such as improved household latrine access.^44^ Though a decline trend observed, high prevalence of annual childhood diarrhoea is still reported in some districts (e.g Metema, Mirab Armacho) (Figure 4).

### Contribution of climatic condition to diarrhoea and cholera outbreaks

In each year, diarrhoea prevalence increased at the beginning of rainy months starting June and in the dry season between January and March. The rainfall events following the dry periods increased diarrhoea incidence because heavy rainfall and runoff can pollute drinking water supplies via transporting pathogens of diarrhoea into unsafe drinking water supplies.^9^ The high prevalence of diarrhoea in dry season might be because of two risk factors: i) increased ambient temperature can favor the growth and survival of bacterial and protozoan pathogens 45; ii) during the dry months, lack of rainfall can lead to water scarcity and lower water quality hence increased accumulation of fecal contamination in the environment.^46^ Furthermore, the impact of elevated ambient temperature on diarrhoea was supported by a study conducted in Asia.^47^

The occurrences of cholera epidemics in the Lake Tana Basin were linked to climate conditions. Similarly, cholera outbreaks following heavy rainfall were also reported in the Great Lakes region of Africa.^48^ For instance, the 2006 cholera outbreak occurred during the heavy rainfall.^41^ The second cholera outbreak started during rainy months following the event of El-Niño in 2015 to 2016 in Ethiopia.^42^, ^43^ Similarly, following El-Niño years, cholera cases increased threefold by 50000 cases in East Africa.49 Furthermore, the impact of El-Niño by elevating water temperature was the major climatic factor for increased cholera incidence.^50^, ^51^

The plausible reasons for contribution of climate changes to cholera outbreaks would be: i) heavy rainfall and runoff can transport V. cholerae from sewage and water bodies into the unsafe drinking water supplies Moreover, *V. cholera* can be acquired from environmental sources as it can survive between outbreaks in the aquatic environment. 52 This conforms to the local expert survey report which stated that the source of cholera outbreaks were holly surface water (Tsebele) sources during rainy monhs; 53 ii) the concentration of *V. cholera* increased in drinking water supplies following heavy rainfall and El Niño;^54^ iii) warm air temperature can increase the growth rate and survival of *V. cholerae*. Hence, climate change and environmental factors can contribute to cholera outbreaks.^55^

The association of cholera to gender and social factors Although biologically *V. cholerae* has an equal probability of causing cholera against gender; females were disproportionately affected by cholera outbreaks. The possible reasons might be women are more responsible for caring cholera patients in the family members as well as they are more engaged in fetching water compared to men.

Social factors such as huge gatherings (example; pilgrimage and celebrations), poor awareness of cholera, lack of sanitation facilities can contribute to the spread of V. cholerae transmission in the community.^53^ For instance, during the 2016, 2017 cholera outbreaks, holly water (Tsebele) sources were the main hotspot areas for the spread of cholera outbreaks because of overcrowding by Orthodox Christian followers as they use hollywater for drinking and bathing with a goal of healing from their illness. The main strength of this systematic review was the inclusion of a four-year retrospective childhood diarrhea cases and cholera outbreak reports from 61 districts. However, one possible limitation was that the four-year secondary data was not included from your 2018 to 2022 due to COVID 19 pandemic and political instability in Ethiopia which could not allow recording and reports from health facilities. In addition, as the secondary data is a health service facility based, the data could not represent the community childhood diarrhoea as children with diarrhoea might not visit health facilities specially during the rainy months.

## Conclusion

Childhood diarrhea is still a major cause of morbidity in children under five years of age in the Lake Tana Basin. The frequently associated risk factors for prevalence of childhood diarrhea were unsafe water supplies and lack of sanitation. Moreover, climate conditions such as heavy precipitations and El-Niño were linked to the episodes of cholera epidemics in the Lake Tana Basin. Intervention measures need to consider seasonal patterns of childhood diarrhea as well as cholera outbreak preparedness should be aware during the events of climate changes.

## Data Availability

Additional data are available on Table S1. Supplementary data.
